# Attitudes Regarding the Use of Ventilator Support Given a Supposed Terminal Condition among Community-Dwelling Mexican American and Non-Hispanic White Older Adults: A Pilot Study

**DOI:** 10.1100/2012/852564

**Published:** 2012-05-02

**Authors:** M. Rosina Finley, Johanna Becho, R. Lillianne Macias, Robert C. Wood, Arthur E. Hernandez, David V. Espino

**Affiliations:** ^1^Department of Family & Community Medicine, The University of Texas Health Science Center at San Antonio, San Antonio, TX 78229, USA; ^2^Department of Psychology, Georgia State University, Atlanta, GA 30302, USA; ^3^College of Education, Texas A&M University at Corpus Christi, Corpus Christi, TX 78412, USA

## Abstract

*Purpose*. To determine the factors that are associated with Mexican Americans' preference for ventilator support, given a supposed terminal diagnosis. *Methods*. 100 Mexican Americans, aged 60–89, were recruited and screened for MMSE scores above 18. Eligible subjects answered a questionnaire in their preferred language (English/Spanish) concerning ventilator use during terminal illness. Mediator variables examined included demographics, generation, religiosity, occupation, self-reported depression, self-reported health, and activities of daily living. *Results*. Being first or second generation American (OR = 0.18, CI = 0.05–0.66) with no IADL disability (OR = 0.11, CI = 0.02–0.59) and having depressive symptoms (OR = 1.43, CI = 1.08–1.89) were associated with preference for ventilator support. *Implications*. First and second generation older Mexican Americans and those functionally independent are more likely to prefer end-of-life ventilation support. Although depressive symptoms were inversely associated with ventilator use at the end of life, scores may more accurately reflect psychological stress associated with enduring the scenario. Further studies are needed to determine these factors' generalizability to the larger Mexican American community.

## 1. Introduction

Use of ventilation at the end of life continues to be a difficult and controversial procedure. While patients may benefit on the short term from ventilation, the long-term benefits in the context of a terminal illness and the impact on quality of life have called the practice into question [1]. Also, families, who are often faced with making these decisions, are often not in the best position to determine patients wishes further complicating the decision to use artificial ventilation [2].

Choices regarding ventilator support are a standard part of discussions with elders or their families regarding end-of-life care. For elder patients this decision is heavily influenced by belief in recovery and the presence of age-related comorbid illnesses that might influence the prognosis. McCarthy and colleagues found that 63% of subjects over age 65 in the Framingham Heart Study said they would rather die than be placed on ventilator support to prolong their life [3]. Furthermore, older adults who are pessimistic and depressed are especially likely to prefer to forego ventilator support in the case of a terminal illness, probably because they do not see themselves as likely breathing again on their own [4].

Cultural beliefs also help to shape these end-of-life attitudes and appear to influence whether individuals agree to an advanced directive that precludes supportive ventilation. It has been observed that African Americans and/or low-income individuals are less likely than non-Hispanic Whites and/or more affluent individuals to prefer extensive life support to include ventilator use [4, 5]. In contrast, Caralis et al. found that in a group of younger Hispanics in the Miami area, they more frequently favored cardiopulmonary resuscitation and ventilator use than either African Americans or Non-Hispanic Whites [5]. Therefore, whether or not ventilator use, especially in the context of a hypothetical terminal condition, is a choice preferred by older Mexican Americans remains an open question.

The purpose of this study was to examine older Mexican Americans attitudes toward mechanical ventilation as a life support option in the context of a hypothetical end-of-life terminal illness within a sample of community-dwelling individuals over age 60.

## 2. Designs and Methods

### 2.1. Setting and Sample

Subjects were recruited between December 2007 and May 2008 at four geographically separate primary care outpatient practices in San Antonio, TX. Recruitment was completed by bilingual (Spanish/English) research assistants in the clinic waiting areas. The study sample included 208 older adults (age 60–89) classified through self-report as either non-Hispanic white or Mexican American, who scored higher than 18 on the Mini Mental State Examination. All subjects provided informed consent and both Institutional Review Boards (the University of Texas Health Science Center and the University of Texas at San Antonio) approved the one-hour interview study. All subjects took the interview in their language of preference (English or Spanish). For purposes of this study, attitudes of the 100 Mexican American subjects were examined.

### 2.2. Outcome Measure

Ventilator support attitudes were assessed by asking subjects to “think about what things would be like if you were diagnosed as having a terminal illness, which means your health could not improve no matter what the doctor does.” Subjects' responses to the question, “would you want to be connected to a machine to help you breathe?” [4] were recorded on a four-point Likert scale ranging from “strongly disagree” to “strongly agree.” Scores were then dichotomized as strongly agree/agree versus strongly disagree/disagree.

### 2.3. Other Variables

Age, gender, education, occupation, and marital status were collected as demographic measures. Preferred language use and generational status were also assessed. Subjects were asked which language they prefer to speak at home. Religiosity was measured using a validated 11-item Likert scale containing statements about intrinsic spiritual beliefs and formal religious motivation [6].

 In addition, subjects were asked if they, their mother, or father had been born in the United States (USA) or Mexico. If all three were US born, the subject was considered third generation. If the subject was born in the USA and either parent was born in Mexico they were considered second generation. Subjects born in Mexico were considered first generation. For purposes of analysis, the first and second generation subjects were combined and contrasted against those in the third generation.

Depressive symptoms were assessed using a 15-item Geriatric Depression Scale and examined as a continuous variable [7, 8]. Instrumental Activities of Daily Living (IADL) function was dichotomized as needing no help with any activity versus needing help with at least one activity [9]. Self-reported health status was measured by asking subjects, “in general, how do you see your health today?” and coded using a 4-point Likert scale ranging from poor (1) to excellent (4). “Poor” was combined with “Fair” health and contrasted against the combination of “good” and “excellent” health.

### 2.4. Statistical Analysis

Logistic regression analysis was used to test associations with predictor and outcome variables using SPSS version 17 (Chicago, IL). Variables were selected for the multiple variable model based on either their hypothesized importance or bivariate associations as analyzed by *t*-test and chi-square with ventilator attitudes and ethnicity.

## 3. Results

The mean age of the sample was 70.1 years. About 58% were female, 62.6% were married, and the majority reported greater than 10 years of education (63.0%). A significant proportion of the sample had been in a professional occupation (34%). English proficiency was high at 75.8% and over a third of the sample were 3rd generation American (34.4%). Health in the sample was good with 4.0% of the sample reporting poor self-reported health and 24% reporting one or more IADL disabilities. Overall depressive symptomatology was also low (mean GDS score = 1.64, SD = 1.95). Religiosity was high in our participants with a mean score of 31.9 (SD = 4.3) out of a possible maximum score of 37.

Table 1 shows the characteristics of those who agreed with ventilator support with 28% of the sample indicated agreement and/or strong agreement for its use at the end of life. Being male (OR = 2.54, CI: 1.02–6.28) and first or second generation Mexican American (OR = 3.94, CI: 0.122–12.66).

In the logistic regression analysis (Table 2) first and second generation Mexican Americans were more likely to agree with ventilation compared with third generation Mexican Americans, (OR = 5.56, CI: 1.51–20.0). Having no IADL disability (Table 3) in a respondent was also associated with an agreement with ventilation (OR = 9.09, CI: 1.695–50). Also, depressive symptoms predicted agreement with ventilator use in the case of a terminal condition in our cohort (OR = 1.43, CI: 1.08–1.89).

## 4. Discussion

Our study is the first of which we are aware to examine attitudes towards ventilator use among older, community-dwelling older Mexican Americans in a hypothetical end-of-life scenario. Older Mexican Americans who were first or second generation American or were functionally independent were more likely to favor end-of-life artificial ventilation. 

In previous studies of older Mexican Americans cultural variables have not been shown to be significant in ethical decision making. Our results indicate those older Mexican Americans who recently immigrated to the United States are more likely to favor aggressive end-of life care. This may indicate a general mistrust of the health care system with a reflexive response to favor aggressive care in terminal situations. It also may reflect the difficulties associated with language differences, especially when dealing with complex and life-ending decisions such as ventilator use. It is possible that assimilation results in older US born Mexican Americans making end-of-life decisions which are more consistent with the majority of Americans when compared to the relatively newer immigrant Mexican Americans [10]. 

Also of interest were that those who were IADL independent were more likely to prefer end-of life ventilation. This is consistent with what has been seen previously in other older populations [11]. Healthier persons usually prefer ventilation in these situations, even in an end-of-life scenario [12]. That the more functionally independent older Mexican American may have difficulty translating his current functional status to that of a sicker, more frail person in a terminal condition is not unexpected.

Depressive symptoms were also noted to be significant and has been seen in multiple studies as a significant factor in choosing ventilator use [11, 13]. The presence of depressive symptoms may ultimately be more important than other factors such as religiosity in understanding older Mexican American decision making with regard to the issue of ventilator use at the end of life. However, the practical difference (1.43 versus 2.18) on the 15-item GDS renders the depressive symptom variation seen in our sample to that of minimal importance from a clinical perspective but may more accurately reflect the psychological stress in working through this hypothetical scenario.

### 4.1. Strengths and Limitations

A strength of the study was the sample of community-dwelling older Mexican Americans with relatively good health and better education than traditionally seen in other Mexican American origin studies. The limitations of this study include the convenience sample strategy and the small sample size which limits generalizability of our results. However, findings from our study do provide an important first impression of older Mexican American's end-of-life ventilator preferences which can provide an evaluative framework for larger studies in the future.

## 5. Conclusions

First and second generation older Mexican Americans and those without IADL disability are more likely to prefer end-of-life ventilation support. Those older Mexican Americans relatively new to the country may have a level in mistrust in the health care system or may not understand the implications of mechanical ventilation due to language barriers. Those with limited disabilities may have difficulty putting themselves into a hypothetical end-of-life scenario. Also, those with depressive symptoms also are more likely to prefer support but these results may more accurately reflect mild psychological stress rather than an actual clinical depression. Sensitivity by physicians to these unique characteristics might result in more satisfactory outcomes when interacting with older Mexican Americans at the end of life.

## Figures and Tables

**Table 1:** Description of the community dwelling older Mexican American sample.

Variables	Mexican American (*n* = 100)
Age, mean ± SD	70.1 ± 7.9

Gender, *n* (%)	
Male	42 (42.0%)
Female	58 (58.0%)

Education, *n* (%)	
Grade 10 or less	37 (37.0%)
10th Grade	63 (63.0%)

Professional/nonprofessional occupation	34 (34.0%)

Health, *n* (%)*	
Good or excellent health	62 (61.6%)
Fair or poor health	37 (38.4%)

GDS depressive symptoms	
Range 0 to 10 (mean, SD)	1.6 (1.95)

IADL disability, *n* (%)	24 (24.0%)

Marital status, *n* (%)*	
Married	62 (62.6%)
Never married, widowed, and separated	37 (37.4%)

Religiosity Scale, mean ± SD	31.9 ± 4.3

English proficiency*	72 (75.8%)
Non-English proficient	

3rd generation American*	32 (34.4%)
1st and 2nd generation	

% agreement with physician assisted suicide*	(30%)

SD: standard deviation; IADL: Instrumental Activities of Daily Living; *: missing values.

**Table 2:** Characteristics associated with agreement with ventilator use in a terminal condition.

	Disagree/strongly disagree	Agree/strongly agree	Unadjusted odds ratio	*P* value
	*n* = 66 (%)	*n* = 28 (%)	(95% CI)	

Age (Mean, SD)	71.1 (8.2)	69.0 (7.1)	0.97 (.91–1.02)	0.24

Gender		
Female	41 (62.1%)	11 (39.3%)
Male	25 (37.9%)	17 (60.7%)	2.54 (1.02–6.28)	0.04

Education		
≤10 years	23 (34.8%)	13 (46.4%)
>10 years	43 (65.2%)	15 (53.6%)	0.62 (0.25–1.51)	0.29

Occupation		
Nonprofessional	45 (70.3%)	15 (53.6%)
Professional	19 (29.7%)	13 (46.4%)	2.05 (0.82–5.13)	0.12

Self-reported health		
Excellent/good	40 (60.6%)	19 (67.9%)
Fair/poor health	26 (39.4%)	9 (32.1%)	0.73 (0.29–1.86)	0.51

GDS				
Depressive symptoms (Mean, SD)	1.43 (2.0)	2.18 (1.7)	1.21 (0.96–1.51)	0.10

*IADL *				
No disability	49 (74.2%)	25 (89.3%)	2.89 (0.773–10.75)	0.10
Any disability	17 (25.8)	3 (10.7%)		

Living spouse	40 (61.5%)	18 (64.3%)	1.125 (0.45–2.82)	0.802
Widowed/not married	25 (38.5%)	10 (35.7%)		

Religiosity scale (mean, SD)	31.4 (4.56)	30.68 (3.88)	0.96 (0.87–1.07)	0.46

Income				
<$20k	41 (63.1%)	15 (53.6%)		
≥$20k	24 (36.9%)	13 (46.4%)	1.48 (0.60–3.63)	0.390

English proficiency				
No	14 (21.2%)	9 (33.3%)		
Yes	52 (78.8%)	18 (66.7%)	0.54 (0.2–1.46)	0.22

1st or 2nd	38 (59.4%)	23 (85.2%)	3.94 (0.122–12.66)	0.017
3rd generation American	26 (40.6%)	4 (14.8%)		

IADL: Instrumental Activities of Daily Living.

**Table 3:** Factors associated with older Mexican Americans' positive attitudes toward Ventilator Support.

	Adjusted OR	CI	*P*
1st or 2nd generation American	5.56	1.51–20.0	0.010
No IADL disability	9.09	1.695–50	0.011
GDS depressive symptoms	1.43	1.08–1.89	0.011

Nagelkerke *R* Square: 0.27; IADL: Instrumental Activities of Daily Living.
